# ﻿A review of the leaf-beetle genus *Sinoluperus* Gressitt & Kimoto, 1963 (Coleoptera, Chrysomelidae, Galerucinae) from China, with the description of a new species

**DOI:** 10.3897/zookeys.1200.116337

**Published:** 2024-05-09

**Authors:** Hai-Dong Yang, Chuan Feng, Xing-Ke Yang

**Affiliations:** 1 Guangdong Public Laboratory of Wild Animal Conservation and Utilization, Guangdong Key Laboratory of Animal Conservation and Resource Utilization, Institute of Zoology, Guangdong Academy of Sciences, Guangzhou, Guangdong 510260, China Institute of Zoology, Guangdong Academy of Sciences Guangzhou China; 2 Key Laboratory of Zoological Systematics and Evolution, Institute of Zoology, Chinese Academy of Sciences, 1 Beichen West Road, Chaoyang District, Beijing 100101, China Institute of Zoology, Chinese Academy of Sciences Beijing China; 3 University of Chinese Academy of Sciences, No. 19(A) Yuquan Road, Shijingshan District, Beijing, 100049, China University of Chinese Academy of Sciences Beijing China

**Keywords:** Key, Nanling mountains, taxonomy

## Abstract

In this study, all species of the leaf-beetle genus *Sinoluperus* Gressitt & Kimoto, 1963 from China are redescribed based on the reexamination of type specimens, and a new species, *S.variegatus***sp. nov.** from Nanling Mountains, is described. A key to the three Chinese species of *Sinoluperus* is provided, as well as photographs of the habiti and aedeagi of these species.

## ﻿Introduction

The genus *Sinoluperus* was established by Gressitt and Kimoto (1963) based on *Sinoluperussubcostatus* Gressitt & Kimoto, 1963 from southern China (Zhejiang, Jiangxi, Sichuan, Guangdong, and Hainan). Not until 1998 was a second species, *Sinoluperuswuyiensis* Yang & Wu, 1998 described from Wuyishan Mountains, China (Fujian) (Yang et al. 1998). Mohamedsaid (1999) described the third species, *Sinoluperusbeta* Mohamedsaid, 1999, from Maxwell’s Hill in Perak, Malaysia. In 2008, Lopatin reported the fourth species, *Sinoluperusvietnamicus* Lopatin, 2008 from Vietnam. Prior to the present study, only four species of *Sinoluperus* have been known, all distributed in the Oriental Region and with three species recorded from China.

*Sinoluperus* species can be identified by following characters: body elongate, medium-sized, dorsal surface of body hairless and mostly yellowish brown or black-brown; head and pronotum about with same width, vertex covered with punctures; frontal tubercles transverse; antennae extendng to apex of elytra or longer than body, antennomere 2 shortest, antennomere 3 approximately 3.5× as long as antennomere 2, antennomere 4 nearly equal in length to antennomere 3 or slightly longer than 3, antennomeres 5–11 decreasing slightly in length; pronotum slightly wider than long, all margins bordered, disc slightly convex, without impression, finely and sparsely covered with punctures; scutellum triangular, with punctures or impunctate; lateral margins of elytra straight and parallel, humeri strongly convex, elytron with longitudinal impressions and punctures along impressions; elytral epipleuron broad at base, narrowed from middle to apex, disappeared before apex; procoxal cavity open behind; tibia with apical spur; first metatrsomere equal to combined remaining tarsomeres; aedeagus ventrally with a flat middle part, base with a large orifice, apex strongly sclerotized and with protrusions; in lateral view, apical protrusions, with flat middle part, and slightly curved basal part; and last visible sternite three-lobed in male and complete in female.

Recently, when we studied leaf beetles from the Nanling Mountains, southern China, two species of *Sinoluperus* were identified: *S.wuyiensis* Yang & Wu, 1998 and *S.variegatus* sp. nov.

## ﻿Materials and methods

The specimens of new species were collected in the Nanling Mountains by net sweeping. Specimens were preserved in 100% ethanol. Morphological characters were examined with an Olympus SZ61 microscope. Male genitalia from each species were dissected using the following procedure: for dried or ethanol preserved specimens, the abdomen was removed from each specimen, boiled in water for 5–10 min, then transferred to a vial containing 10% KOH solution. The abdomen with the aedeagus was washed in distilled water 3 or 4 times, transferred to a cavity slide using fine forceps. There aedeagus was separated from the abdomen using a hooked, fine dissecting needle. Habitus images were taken using a Canon 5DSR/Nikon SMZ25 digital camera. Aedeagus images were taken using a Nikon D610 digital camera, attached to a Zeiss V/A1 microscope (with 5× objective lens). A cable shutter release was used to prevent the camera from vibration. To obtain the full depth of focus, all images were stacked using Helicon Focus 7 and the resulting output was edited with Adobe Photoshop CC.

The type specimens of new species are deposited in the following two instititions: the Institute of Zoology, Guangdong Academy of Sciences, Guangzhou, China (**IZGAS**); Institute of Zoology, Chinese Academy of Sciences, Beijing, China (**IZAS**).

Abbreviations and depositories used in the paper:

**TL** type locality

**TD** type deposition

**CAS**California Academy of Sciences, San Francisco, California, USA

**IZAS** Institute of Zoology, Chinese Academy of Sciences, Beijing, China

**IZGAS** Institute of Zoology, Guangdong Academy of Sciences, Guangzhou, China

## ﻿Taxonomic account

### ﻿Key to Chinese species of *Sinoluperus*

**Table:** 

1	Vertex covered with sparsed punctures (Fig. 1A); aedeagus slender, two apical protrusions strongly produced apically, long, cone-shaped, close to each other	***S.variegatus* sp. nov.**
–	Vertex covered with dense punctures (Fig. 1B, C); aedeagus robust, two apical protrusions weakly developed, short, nipple-shaped or short cone-shaped	**2**
2	Pronotum ~1.4× as wide as long; elytra black-brown or yellowish brown	***S.subcostatus***
–	Pronotum twice as wide as long, elytra yellowish-brown	***S.wuyiensis***

#### ﻿ Sinoluperus variegatus sp. nov.

Taxon classificationAnimaliaColeopteraChrysomelidae

E6BB099D-EBE7-5652-87FB-A1A8D04095EF

https://zoobank.org/63019284-6F92-46C7-8FA9-AEDFB2B2E8B8

Figs 1A , 2 , 3 , 4

##### Type material.

***Holotype***: ♂ (Fig. 2), China, Hunan Province, Yizhang, Mangshan National Nature Reserve, zeziping, 22 May 2021, Nanling investigation team leg., IZGAS. ***Paratypes***: 6♂♂1♀, same data as for preceding. 1♀, China, Guangdong Province, Ruyuan, Nanling National Nature Reserve, 20 May 2021, Nanling investigation team leg., IZGAS. 1♂, same data as for preceding, 29 May 2021, Chuan Feng leg., IZGAS. 1♂1♀, same data as for preceding, 11 Jun. 2021, Chuan Feng et al. leg., IZGAS. 3♀♀, same data as for preceding, 19 Jun. 2021, Chuan Feng et al. leg., IZGAS.

**Figure 1.**
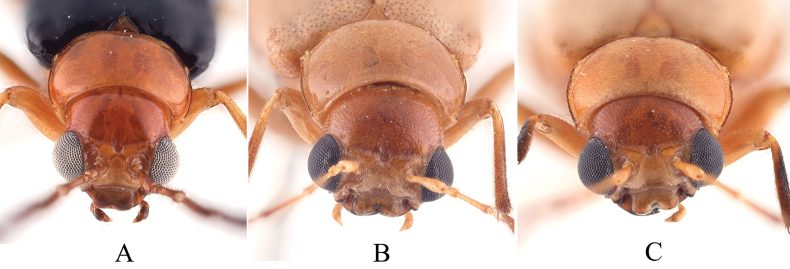
Head of *Sinoluperus* species **A***S.variegatus* sp. nov. **B***S.subcostatus* Gressitt & Kimoto, 1963 **C***S.wuyiensis* Yang & Wu, 1998.

**Figure 2.**
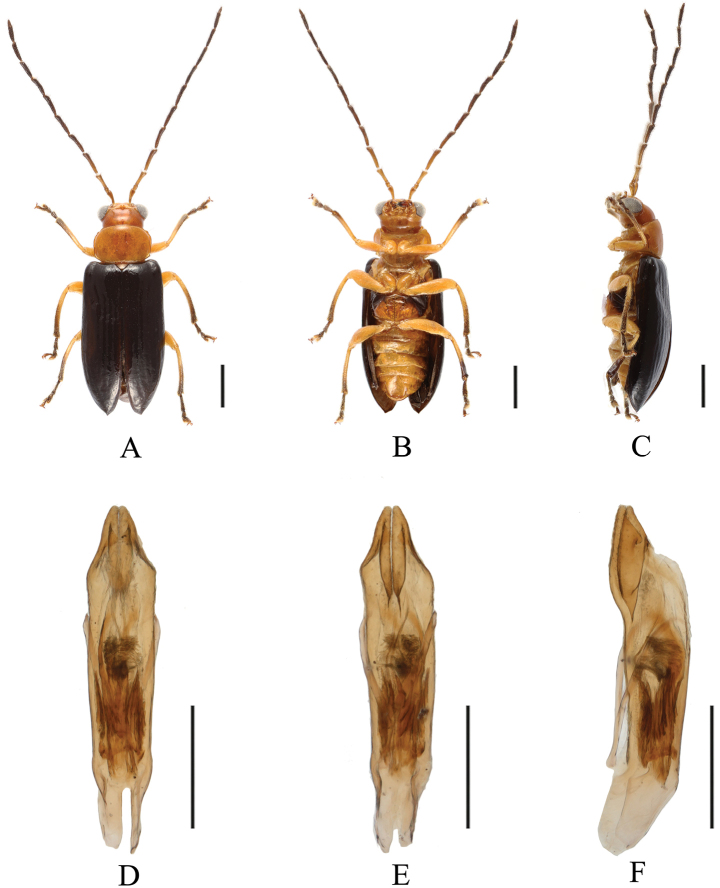
*Sinoluperusvariegatus* sp. nov. (holotype, male) **A–C** habitus (male) **D–F** aedeagus **A, D** dorsal view **B, E** ventral view **C, F** lateral view. Scale bars: 1 mm (**A–C**); 0.5 mm (**D–F**).

**Figure 3.**
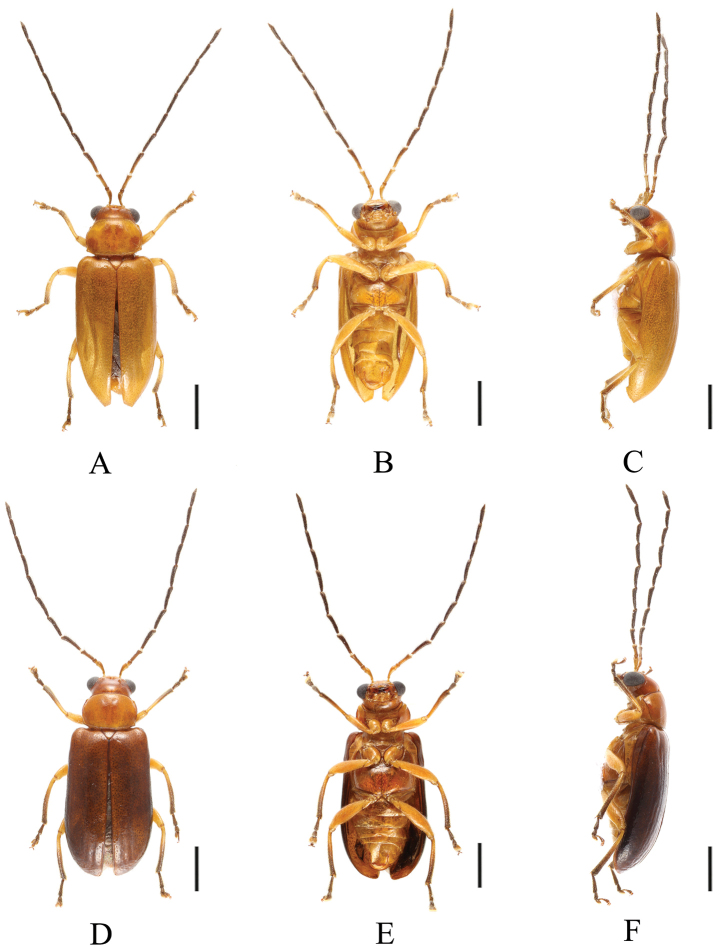
*Sinoluperusvariegatus* sp. nov. (paratype male) **A–F** habitus **A, D** dorsal view **B, E** ventral view **C, F** lateral view. Scale bars: 1 mm.

**Figure 4.**
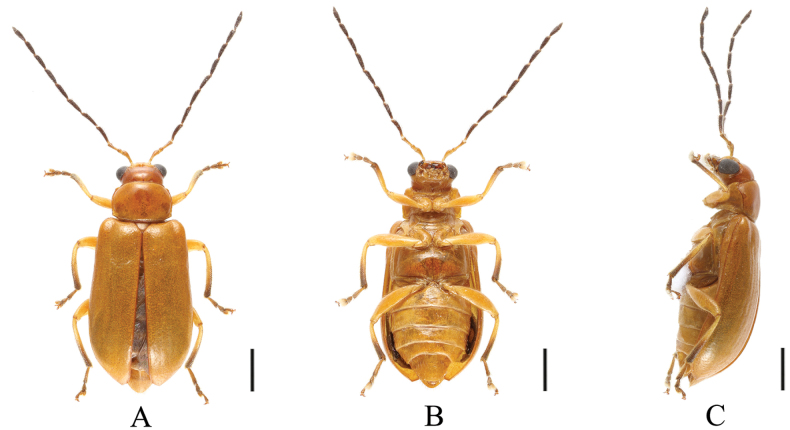
*Sinoluperusvariegatus* sp. nov. (paratype, female) **A–C** habitus **A** dorsal views **B** ventral views **C** lateral views. Scale bars: 1 mm.

##### Diagnosis.

The new species closely resembles *S.subcostatus* Gressitt & Kimoto, 1963, but it differs from the latter by its slender aedeagus with a gradually narrowed apical part in ventral view. In *S.subcostatus*, the aedeagus is robust, and its apical part is abruptly narrowed in ventral view. Light-colored specimens of new species closely resemble *S.wuyiensis* Yang & Wu, 1998. However, the vertex of *S.variegatus* sp. nov. almost impunctate, vertex of *S.wuyiensis* strongly and closely punctate.

##### Description.

**Male.** Length: 4.5–5.0 mm. Head, pronotum, scutellum, ventral side of body, femur, and basal half of tibia yellow or orange. Antennae black-brown, with antennomeres 1–3 yellow-brown, most of metepimeron brown. Elytra black-brown or pale in some specimens. Apical half of tibia, tarsus, and claw black-brown.

Vertex with sparse punctures, with fine reticulation. Frontal tubercles transverse, extending downward between antennal bases. Antennae longer than body; antennomeres 1 bare, rod-shaped, antennomeres 2–11 with short hairs, antennomere 2 shortest, antennomere 3 ~3.5× as long as antennomere 2; antennomere 4 ~1.3× as long as antennomere 3, thick and curved at apex, antennomere 5 equal to antennomere 4 in length, antennomeres 6–11 gradually shortened.

Pronotum ~1.5× as wide as long, lateral margins straight at base and slightly rounded at apex; three setae present on each side of lateral margins, basal margin slightly convex, anterior margin slightly concave, anterior and posterior angles thickened and rounded; disc strongly convex, with sparse and fine punctures, shiny.

Scutellum triangular, smooth, impunctate.

Elytra wider than pronotum at base, humeri strongly convex, lateral sides subparallel and gradually widened posteriorly. Disc with 10 shallow longitudinal grooves, covered with small punctures in grooves, interstices of punctures wider than diameter of individual puncture. Elytral epipleuron broad at base, narrowed at middle, gradually narrowed from middle to apex.

Legs strong, each tibia with distinct spur at apex, tarsomere 1 of hind tarsi equal to combined remaining tarsomeres.

Aedeagus slender; in ventral view, sides abruptly narrowed near apex, with apical protrusions forming two cones close to each other.

**Female.** Length 6.5 mm. Head, pronotum, scutellum, ventral surface of body, femur, and base of tibia yellowish brown; apical half of tibia, tarsus, claw black brown. Antennae black-brown, with antennomeres 1–3 yellow-brown. Antenna ~2/3 of body length, antennomere 3 ~3× as long as antennomere 2; antennomere 4 ~1.5× as long as antennomere 3, antennomere 5 equal in length to antennomere 4, antennomere 6 slightly shorter than antennomere 5, antennomeres 7–11 gradually widened. Punctures Barely visible on pronotum and elytra.

##### Distribution.

China: Zhejiang, Hunan, Guangdong.

##### Etymology.

The species name (Latin, meaning “variegated”) refers to the variable color of elytra.

#### ﻿ Sinoluperus subcostatus

Taxon classificationAnimaliaColeopteraChrysomelidae

Gressitt & Kimoto, 1963

199420FE-92F5-517C-9148-83C7E5A5856D

**Figure 5.**
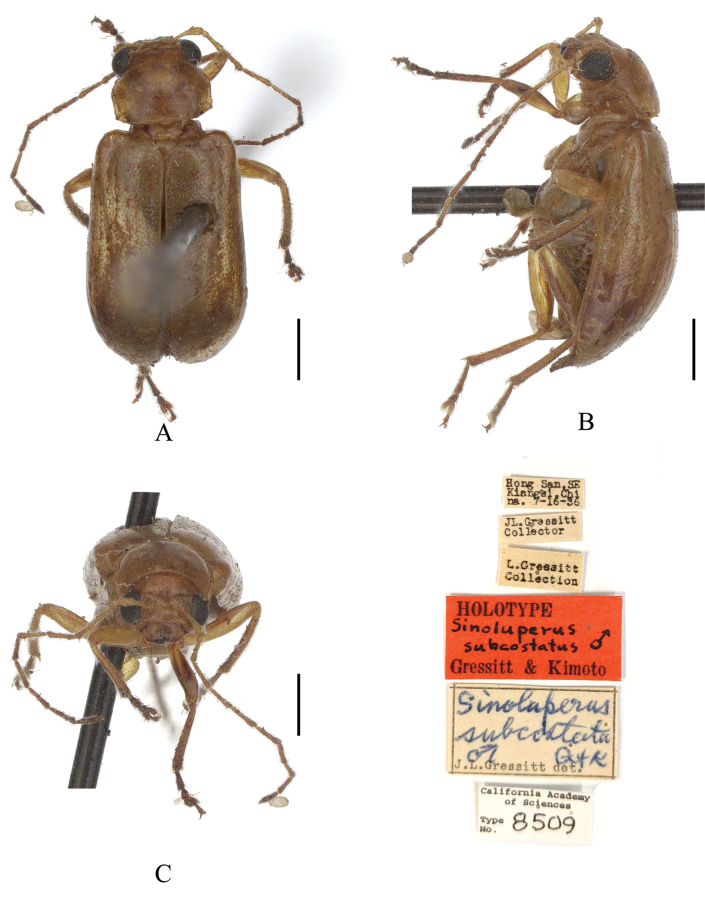
*Sinoluperussubcostatus* (male) (holotype) **A–C** habitus **A** dorsal view **B** lateral view **C** head view. Scale bars: 1 mm.

**Figure 6.**
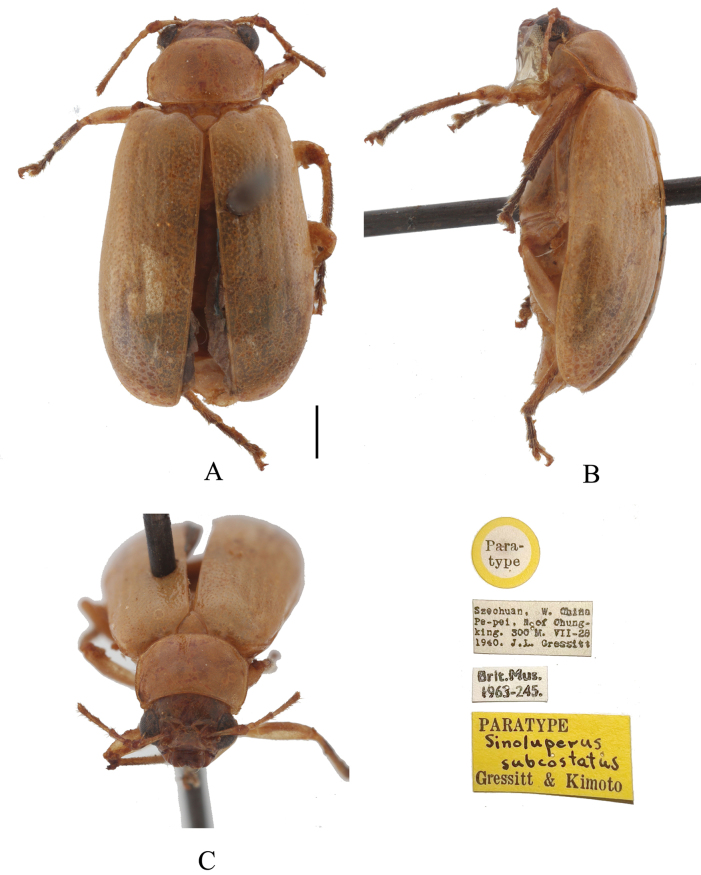
*Sinoluperussubcostatus* (female) (paratype) **A–C** habitus **A** dorsal view **B** lateral view **C** head view. Scale bars: 1 mm.

**Figure 7.**
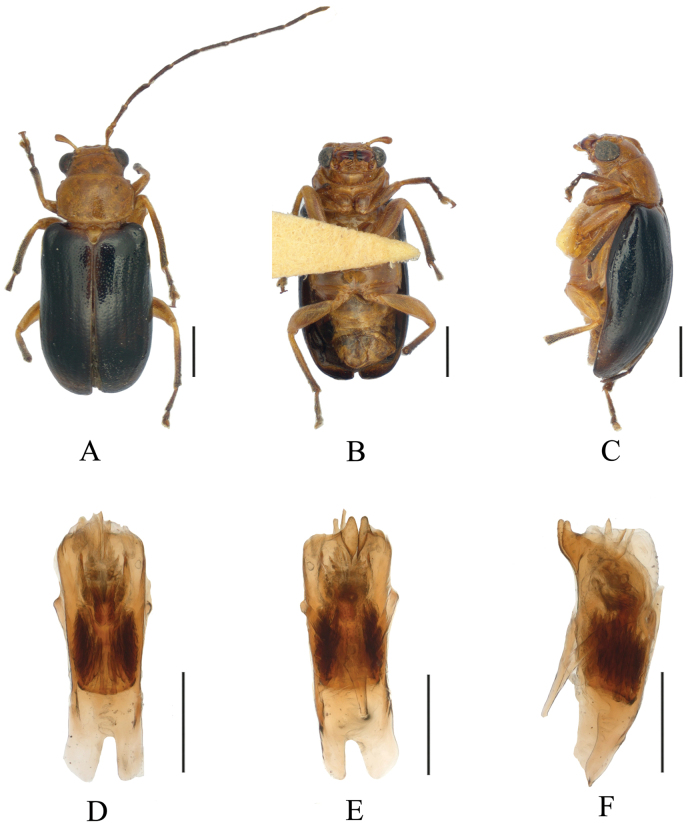
*Sinoluperussubcostatus* (male) (paratype) **A–C** habitus **D–F** aedeagus **A, D** dorsal view **B, E** ventral view **C, F** lateral view. Scale bars: 1 mm (**A–C**); 0.5 mm (**D–F**).

**Figure 8.**
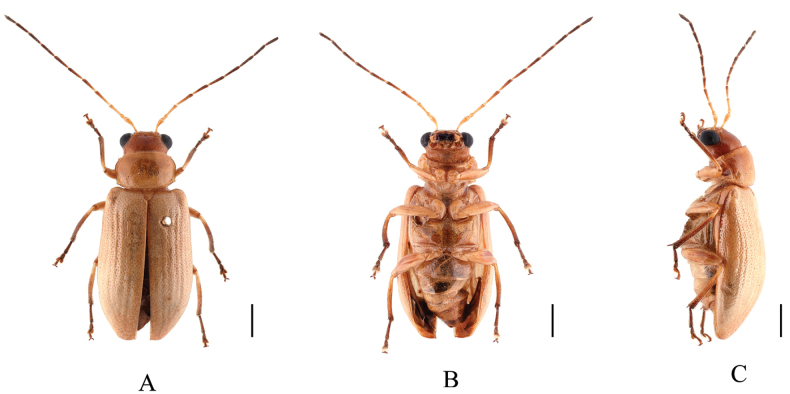
*Sinoluperussubcostatus* (female) **A–C** habitus **A** dorsal view **B** ventral view **C** lateral view. Scale bars: 1 mm.

Figs 1B , 5 , 6 , 7 , 8

Sinoluperus subcostatus Gressitt and Kimoto 1963: 584. TL: China, Jiangxi. TD: CAS.

##### Type specimens examined.

***Holotype***: ♂. Hong San, SE Kiangsi Prov, China, 16. Jul. 1936, Gressitt leg., CAS8509. ***Paratypes***: ♀. Szechuan, W. China, Pe-pei,, 28. Jul. 1940, Gressitt; 300 m a. s. l. Brit. Mus. 1963-245. ♂. China, Hainan Province, Tai-pin (Dwa-bi), 325 m a. s. l., 22 Jul. 1935, Gressitt leg., IZAS.

##### Additional specimens examined.

1♀, China, Hunan Province, Yizhang, Mangshan National Nature Reserve, chawanggu, 25 Aug 2020, Siyuan Xu leg., IZGAS.

##### Description.

**Male.** Length 4.8–5.2 mm. Body ochraceous, apical half of tibia, tarsus, and claw reddish brown. In the paratype in IZCAS, head, pronotum, scutellum, ventral surface of body, femur, and base of tibia yellow; antennae black-brown with antennomeres 1 and 2 yellow, elytra black-brown with reddish brown at apex. Apical half of tibia, tarsus, and claw brown.

Vertex covered with closed punctures. Frontal tubercles transverse. Antennae longer than body. Antennomeres 1 bare, rod-shaped, antennomeres 2–11 with short hairs, antennomere 2 shortest, antennomere 3 ~3.7× as long as antennomere 2; antennomere 4 ~1.4× as long as antennomere 3, antennomeres 5–11 equal in length, and slightly shorter than antennomere 4.

Pronotum ~1.4× as wide as long, anterior margin straight; basal margin slightly convex, lateral margins straight at base and slightly rounded at apex, anterior angle projecting, basal angle obtuse, disc convex, with sparse punctures.

Scutellum triangular, with several small punctures.

Elytra wider than pronotum basally, humeri strongly convex, lateral margins of elytra gradually widened posteriorly. Elytra disc with 10 shallow longitudinal grooves and covered with small punctures, the interstices of punctures equal with diameter of individual puncture. Epipleuron broad basally, strongly narrowed at middle, gradually narrowed from middle to apex. Leg strong, each tibia with a distinct spur at apex.

Aedeagus robust, in ventral view, with sides slightly dilating near apex; apical protrusions short-cone-shaped, close to each other.

**Female.** Length 5.2–5.5 mm. Head reddish brown; Antennae reddish brown with antennomeres 1–3 yellow, pronotum, scutellum, ventral surface of body, femur, and base of tibia brown or yellowish brown; apical half of tibia, tarsus, claw reddish brown.

##### Distribution.

China: Zhejiang, Jiangxi, Hongkong, Guangdong, Hainan, Sichuan; Laos.

#### ﻿ Sinoluperus wuyiensis

Taxon classificationAnimaliaColeopteraChrysomelidae

Yang & Wu, 1998

8EF46B76-F58B-56A5-A2E6-16D7F8AD620D

Figs 1C , 9 , 10

Sinoluperus wuyiensis Yang & Wu, 1998: 262. TL: China, Fujian. TD: IZAS.

##### Type specimens examined.

***Holotype***: ♂, China, Fujian province, Mount Wuyi, Maopai, 1 Aug. 1997, Yanyu Wu leg., IZAS. ***Paratypes***: 1♂, China, Fujian Province, Mount Wuyi, Pikeng, 520 m a. s. l., 31 Jul. 1997, Yanyu Wu leg., IZAS. 2♂♂, Fujian Province, Mount Wuyi, Huangxizhou, 650 m a. s. l., 6 Aug. 1997, Jiashe Wang leg., IZAS. 1♂, China, Fujian Province, Mount Wuyi, Diaoqiao, 540 m a. s. l., 11 Aug. 1997, Youwei Zhang leg., IZAS.

**Figure 9.**
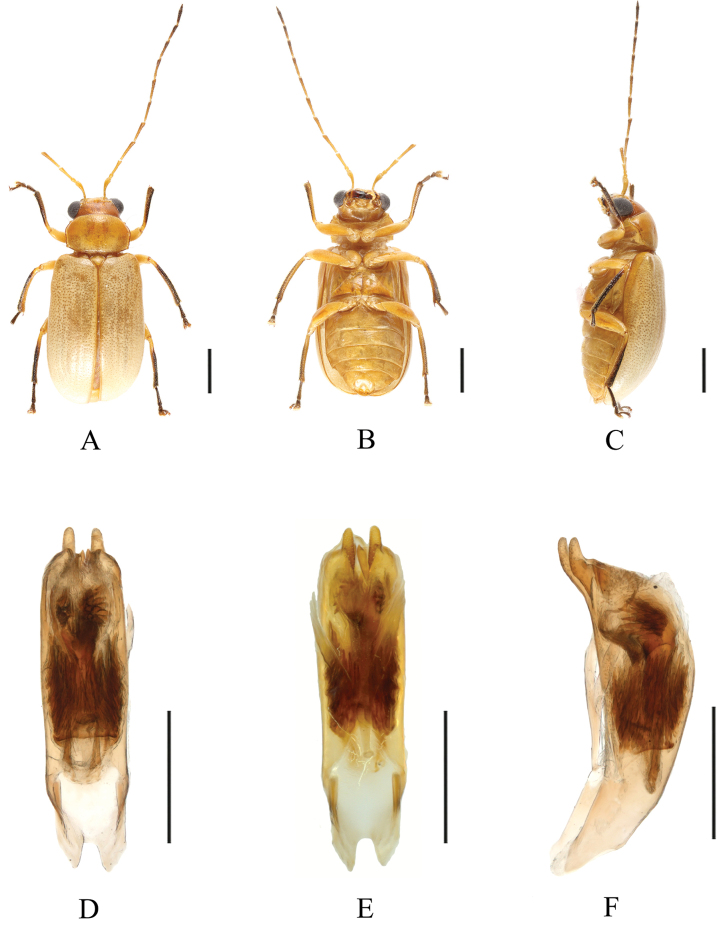
*Sinoluperuswuyiensis* (male) **A–C** habitus **D–F** aedeagus **A, D** dorsal view **B, E** ventral view **C, F** lateral view. Scale bars 1 mm (**A–C**); 0.5 mm (**D–F**).

**Figure 10.**
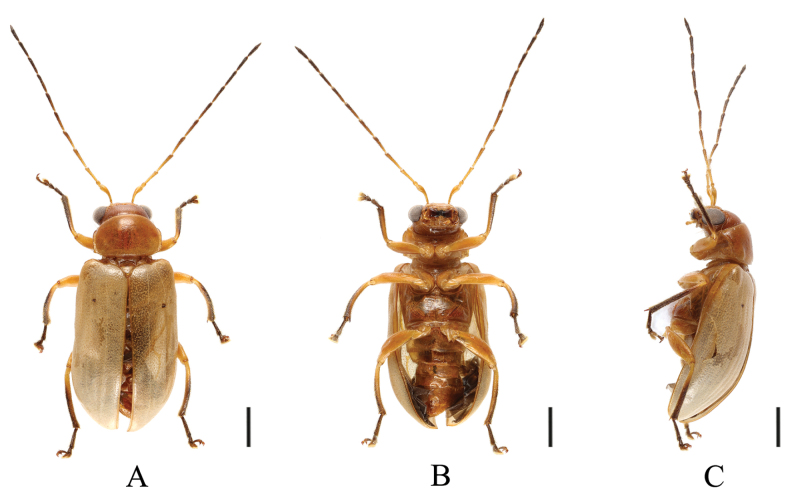
*Sinoluperuswuyiensis* (female) **A–C** habitus **A** dorsal view **B** ventral view **C** lateral view. Scale bars: 1 mm.

##### Additional specimens examined.

1♀, China, Guangdong Province, Ruyuan, Nanling National Nature Reserve, xiaohuangshan, 18 Jul. 2022, Meiying Lin et al. leg., IZGAS. 1♂, China, Guangdong Province, Chebaling National National Nature Reserve, Shixing County, 24 Jun. 2022, Meiying Lin et al. leg., IZGAS.

##### Description.

(♂) Length 4.5–6.0 mm. Head, pronotum, scutellum, ventral surface of body, femur, and base of tibia reddish brown or yellowish brown; antennae ranged from black to brown with antennomeres 1–3 yellow; in some specimens antennae yellow with antennomeres 7–11 black-brown. Elytra yellow; apical half of tibia, tarsus, and claw brown.

Vertex covered with closed punctures. Frontal tubercles small, antennae longer than body. Antennomere 1 bare, rod-shaped, antennomeres 2–11 with short hairs, antennomere 2 shortest, antennomere 3 ~3× as long as antennomere 2; antennomere 4 ~1.2× as long as antennomere 3, antennomeres 4–11 equal in length.

Pronotum ~2× as wide as long, basal and apical margins slightly convex, disc strongly convex with dense punctures.

Scutellum triangular, smooth, impunctate.

Elytra wider than pronotum basally, humeri strongly convex, subparallel-sided but gradually widened posteriorly. Disc with 8–10 longitudinal grooves, covered with dense punctures, interstices of punctures narrower than diameter of individual puncture. Elytral epipleuron broad at base, strongly narrowed at middle, gradually narrowed from middle to apex.

Legs strong, each tibia with distinct spur at apex, segment 1 of hind tarsi equal to combined remaining segments.

Aedeagus robust, with parallel sides and rounded apex in ventral view. Apical protrusions nipple nipple-shaped, small, well separated from each other.

**Female.** Length 5.5 mm. Antennomere 3 ~3.5× as long as antennomere 2; apical ventrite with longitudinal concave in the middle.

##### Distribution.

China: Hunan, Fujian, Guangdong.

## Supplementary Material

XML Treatment for Sinoluperus variegatus

XML Treatment for Sinoluperus subcostatus

XML Treatment for Sinoluperus wuyiensis

## ﻿Additional information

### Author ORCIDs

Hai-Dong Yang https://orcid.org/0000-0002-3157-2090

Chuan Feng https://orcid.org/0009-0001-9299-3526

### Data availability

All of the data that support the findings of this study are available in the main text.
